# Postoperative hypoalbuminemia, albumin supplementation, and short-term complications after radical gastrectomy in elderly gastric cancer patients

**DOI:** 10.3389/fmed.2026.1978269

**Published:** 2026-10-28

**Authors:** Yu-Nan Guan, Xiao-Ming Lin, Xue-Lian Liu, Yu-Chen Guo, He-Lei Wang

**Affiliations:** Department of Gastrocolorectal Surgery, General Surgery Center, The First Hospital of Jilin University, Changchun, Jilin, China

**Keywords:** albumin supplementation, gastric cancer, hypoalbuminemia, perioperative period, postoperative complications

## Abstract

**Objective:**

To examine the association between early postoperative hypoalbuminemia and complications within 30 days after radical gastrectomy in older patients with gastric cancer, and to further explore the association between postoperative albumin supplementation and short-term outcomes among patients who developed hypoalbuminemia.

**Methods:**

This single-center retrospective cohort study included 378 elderly patients who underwent radical gastrectomy for gastric cancer at the First Hospital of Jilin University from January to December 2023. Patients were classified into a hypoalbuminemia group (<30 g/L) and a normal albumin group (>=30 g/L) according to the lowest serum albumin level within the first 7 postoperative days. The primary outcome was Clavien-Dindo grade >=3 complications within 30 days after surgery, and the secondary outcome was Clavien-Dindo grade >=2 complications and postoperative length of stay. Covariates in the main adjusted models were prespecified on the basis of clinical relevance. Firth penalized logistic regression was used for the primary outcome because of the limited number of events, whereas conventional logistic regression was used for Clavien–Dindo grade ≥2 complications. Postoperative nadir albumin was also examined as a continuous variable. Among patients with hypoalbuminemia, propensity score matching was used to explore the association between postoperative albumin supplementation and short-term outcomes. A POD1 landmark sensitivity analysis was additionally performed to reduce temporal overlap between postoperative albumin assessment and complication occurrence.

**Results:**

Among the 378 patients, 246 were classified into the hypoalbuminemia group and 132 into the normal albumin group. The incidence of Clavien–Dindo grade ≥3 complications was higher in the hypoalbuminemia group than in the normal albumin group (15.9% vs. 2.3%, *P* < 0.001). After adjustment for age, body mass index, American Society of Anesthesiologists physical status, preoperative serum albumin, surgical approach, surgical method, intraoperative blood transfusion, and pathological TNM stage, postoperative hypoalbuminemia remained associated with Clavien–Dindo grade ≥3 complications (adjusted OR, 4.51; 95%CI, 1.55–17.54; *P* = 0.004). When postoperative nadir albumin was modeled continuously, each 5-g/L decrease was associated with an adjusted OR of 4.71 (95%CI, 2.43–9.51; *P* < 0.001). The incidence of Clavien–Dindo grade ≥2 complications was also higher in the hypoalbuminemia group (61.8% vs. 24.2%, *P* < 0.001). The corresponding adjusted ORs were 3.75 (95%CI, 2.16–6.62; *P* < 0.001) for the categorical model and 4.21 (95%CI, 2.54–7.21; *P* < 0.001) per 5-g/L decrease in postoperative nadir albumin. Postoperative length of stay was longer in the hypoalbuminemia group [6.0 (5.0–9.0) d vs. 5.0 (4.0–6.0) d, *P* < 0.001]. In the POD1 landmark sensitivity analysis, the associations were attenuated. Lower POD1 serum albumin remained associated with Clavien–Dindo grade ≥2 complications in both the categorical model (adjusted OR, 1.90; 95%CI, 1.09–3.34; *P* = 0.024) and the continuous model (adjusted OR per 5-g/L decrease, 2.08; 95%CI, 1.36–3.22; *P* < 0.001), whereas the associations with grade ≥3 complications were no longer statistically significant. After 1:1 propensity score matching, 154 patients with hypoalbuminemia were included, with 77 patients in each supplementation group. Conditional logistic regression accounting for the matched-pair structure showed an association between recorded postoperative albumin supplementation and Clavien–Dindo grade ≥2 complications (OR, 2.00; 95%CI, 1.12–3.58; *P* = 0.020).

**Conclusions:**

Lower serum albumin levels during postoperative days 1–7 were associated with Clavien–Dindo grade ≥3 and grade ≥2 complications within 30 days after radical gastrectomy in older patients with gastric cancer. However, these associations were attenuated in the POD1 landmark sensitivity analysis, particularly for grade ≥3 complications, highlighting the potential influence of temporal overlap and reverse causation. Because the albumin assessment window overlapped with the complication surveillance period, the main findings should be interpreted as associations rather than evidence of temporal sequence or causality. Among patients with hypoalbuminemia, recorded postoperative albumin supplementation was not associated with a lower incidence of complications; this finding may have been influenced by confounding by indication, residual confounding, and reverse causation. Postoperative assessment should therefore consider surgical trauma, inflammatory response, volume status, and overall recovery rather than relying on a single serum albumin value.

## Introduction

Human serum albumin is the most abundant protein in plasma. It is primarily synthesized by the liver, with normal physiological concentrations maintained at 35–45 g/L, and plays a crucial role in maintaining plasma colloid osmotic pressure, antioxidant defense, and drug transport (1). However, hypoalbuminemia is highly prevalent during the perioperative period after major abdominal surgery and is commonly defined as a serum albumin level <30 g/L. Unlike chronic malnutrition, the pathogenesis of postoperative acute hypoalbuminemia is more complex. Surgery-induced inflammation may disrupt the endothelial glycocalyx and increase capillary permeability, thereby promoting the transvascular escape of albumin into the interstitial space. In addition, the dilutional effect caused by substantial intraoperative fluid resuscitation further contributes to the rapid decline in serum albumin levels (2–4).

A large body of previous evidence has demonstrated that hypoalbuminemia is closely associated with various adverse postoperative outcomes, including surgical site infection, anastomotic leakage, venous thromboembolism, and increased mortality (2, 5–9). Accordingly, exogenous albumin is frequently administered in clinical practice to correct postoperative hypoalbuminemia in the expectation of improving postoperative outcomes. Nevertheless, the effectiveness of this strategy remains controversial. Large randomized controlled trials, including SAFE, SOAP, ALBIOS, and EARSS, as well as several meta-analyses, have shown that routine albumin supplementation does not consistently confer survival benefits or reduce postoperative complications in broad populations of critically ill or mixed surgical patients (10–13). These findings raise the question of whether postoperative hypoalbuminemia represents a modifiable clinical factor or simply reflects surgical trauma, inflammatory responses, and overall postoperative severity in specific patient populations. This uncertainty is particularly relevant in elderly patients with gastric cancer. First, radical gastrectomy involves extensive lymph node dissection and gastrointestinal reconstruction, which are associated with substantial wound exudation and third-space fluid loss; therefore, the causes of postoperative albumin decline are multifactorial in this population (14, 15). Second, elderly patients often have reduced hepatic synthetic reserve and impaired physiological compensatory capacity, making them more vulnerable to alterations in colloid osmotic pressure associated with hypoalbuminemia and potentially contributing to susceptibility to postoperative complications (16–18). However, current evidence regarding postoperative hypoalbuminemia and albumin supplementation is largely derived from heterogeneous mixed populations, and studies focusing specifically on elderly patients undergoing radical gastrectomy for gastric cancer remain relatively limited.

Therefore, this retrospective cohort study investigated the association between postoperative hypoalbuminemia, defined as the lowest serum albumin concentration during postoperative days 1–7, and postoperative complications within 30 days after radical gastrectomy in older patients with gastric cancer. In addition, among patients who developed postoperative hypoalbuminemia, we explored the association between recorded postoperative albumin supplementation and short-term outcomes using propensity score matching. Because the postoperative albumin assessment window partly overlapped with the outcome observation period and albumin supplementation was not randomly assigned, these analyses were intended to evaluate associations rather than treatment effects or causal relationships.

## Materials and methods

### Study design

This was a single-center retrospective cohort study. Clinical data were retrospectively collected from patients who underwent radical gastrectomy for gastric cancer at the First Hospital of Jilin University between January 2023 and December 2023. A total of 400 patients were initially screened. Of these, 8 patients with another primary malignancy, 7 who underwent combined organ resection, and 7 for whom the primary exposure or 30-day postoperative outcome data were unavailable were excluded, leaving 378 patients in the final cohort. The patient selection process is shown in Figure 1. The study was conducted in accordance with the Declaration of Helsinki and reported in accordance with the Strengthening the Reporting of Observational Studies in Epidemiology (STROBE) statement. The study protocol was approved by the Ethics Committee of the First Hospital of Jilin University (approval No. AF-IRB-030-07). Because this retrospective study was based on routinely collected clinical data, the ethics committee waived the requirement for written informed consent. All patient data were de-identified before statistical analysis.

**Figure 1:**
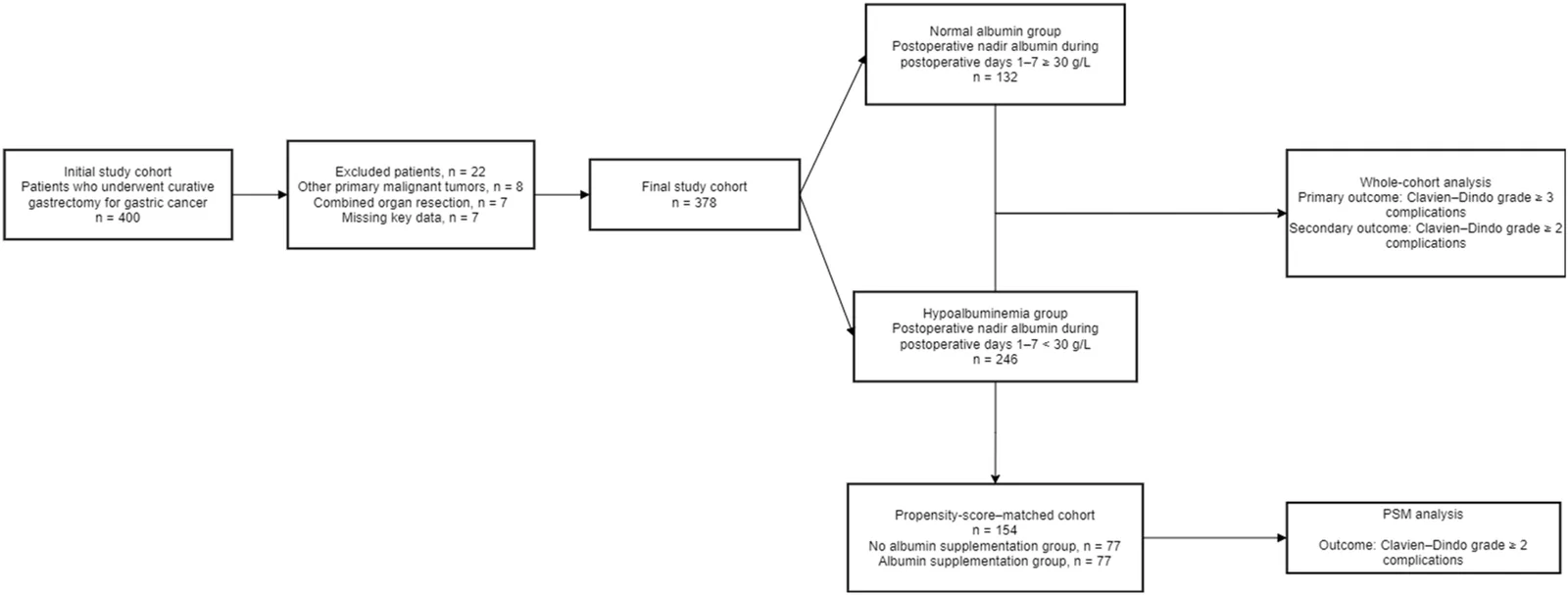
Flowchart of patient selection and statistical analyses. Postoperative nadir serum albumin was defined as the lowest serum albumin level measured during postoperative days 1–7. The whole-cohort analysis evaluated the associations of postoperative nadir serum albumin with Clavien–Dindo grade ≥3 and grade ≥2 complications within 30 days after surgery. In patients with hypoalbuminemia, propensity-score matching was further used to examine the association between albumin supplementation and Clavien–Dindo grade ≥2 complications. PSM, propensity-score matching; C-D, Clavien–Dindo.

### Inclusion and exclusion criteria

The inclusion criteria were as follows: (1) patients aged ≥60 years who were diagnosed with gastric cancer between January 2023 and December 2023; (2) patients who underwent radical gastrectomy for gastric cancer; and (3) patients who had at least one serum albumin measurement within the first 7 postoperative days.

The exclusion criteria were as follows: (1) presence of another primary malignancy; (2) combined organ resection during surgery; and (3) substantial missing perioperative data that precluded ascertainment of the primary exposure or outcome measures. The patient screening process and final cohort are presented in Figure 1.

### Grouping and definition of exposure

The primary exposure in this study was early postoperative hypoalbuminemia. According to the lowest serum albumin level within the first 7 postoperative days, patients in the full cohort were divided into the hypoalbuminemia group and the normal albumin group. Patients with a lowest serum albumin level <30 g/L within 7 days after surgery were classified into the hypoalbuminemia group, whereas those with a lowest serum albumin level ≥30 g/L were classified into the normal albumin group (19). Postoperative days 1–7 were used as the assessment window for albumin status, and no fixed index time point was specified. Serum albumin results were obtained from routine laboratory tests performed during hospitalization. The frequency of albumin testing was determined according to clinical needs and therefore could vary among patients during postoperative days 1–7. Because the analysis was based on retrospective medical records and laboratory data, the database did not contain information on the analyzer model or assay method, and these technical laboratory details could not be reported. Among patients in the hypoalbuminemia group, further stratification was performed according to whether postoperative albumin supplementation was administered. Patients who received intravenous human serum albumin preparations at any time within the first 7 postoperative days were defined as the albumin supplementation group, whereas those who did not receive albumin supplementation were defined as the non-supplementation group. The human albumin preparation used in clinical practice contained 10 g per bottle. Because this was a retrospective study, the initiation, dose, and duration of albumin supplementation were determined by the operating surgeon or attending physician on the basis of clinical judgment rather than a standardized formula. The database did not systematically capture the exact time of first supplementation, cumulative dose, treatment duration, or specific clinical indication for all patients. Accordingly, this study evaluated only the association between recorded albumin supplementation and short-term outcomes and did not assess the efficacy of a standardized intervention.

### Variables and outcome definitions

The primary outcome of this study was Clavien–Dindo grade ≥3 complications occurring within 30 days after radical gastrectomy (20). The secondary outcomes were the occurrence of postoperative complications of Clavien–Dindo grade ≥2 within 30 days after surgery and postoperative length of hospital stay. These outcomes were obtained from inpatient medical records, imaging examinations, laboratory tests, and follow-up records within 30 days after surgery to ensure diagnostic accuracy and completeness. For patients who developed multiple complications, the highest Clavien–Dindo grade within 30 days after surgery was recorded as the final complication grade.

To account for potential confounding, demographic characteristics, preoperative clinical data, surgery-related variables, and pathological characteristics were systematically collected. Covariates included in the main adjusted models were selected on the basis of clinical relevance and their potential relationships with both the exposure and outcomes; the specific variables are described in the Statistical Analysis section. In the subgroup of patients with hypoalbuminemia, the same outcome definitions were used to further explore the association between postoperative albumin supplementation and short-term clinical outcomes. Importantly, postoperative human albumin administration for correction of hypoalbuminemia alone was not considered a postoperative complication and did not, by itself, result in classification as Clavien–Dindo grade II. Albumin supplementation was recorded separately as an exposure variable.

### Statistical analysis

All statistical analyses were performed using R software, version 4.4.2. Continuous variables were expressed as mean ± standard deviation or median with interquartile range, depending on their distribution. Between-group comparisons were performed using the independent-samples t test, Welch's t test, or the Wilcoxon rank-sum test, as appropriate. Categorical variables were expressed as counts and percentages, and between-group comparisons were performed using the chi-square test or Fisher's exact test. All statistical tests were two-sided, and a *P* value <0.05 was considered statistically significant.

### Full cohort analysis

In the full-cohort analysis, patients were divided into the hypoalbuminemia group and the normal albumin group according to the lowest serum albumin level within the first 7 postoperative days. Demographic characteristics, preoperative clinical data, surgery-related variables, pathological characteristics, and postoperative outcomes were compared between the two groups.

Regression models were used to examine the association between early postoperative hypoalbuminemia and complications within 30 days after surgery. Covariates in the main adjusted models were prespecified before analysis on the basis of clinical relevance and their potential confounding effects and were not selected according to *P* values from univariable analyses. The fixed main adjusted model included age, body mass index, American Society of Anesthesiologists physical status classification, preoperative serum albumin, surgical approach, surgical method, intraoperative blood transfusion, and pathological TNM stage.

For the primary outcome of Clavien–Dindo grade ≥3 complications, Firth penalized logistic regression was used because the number of outcome events was relatively limited and only a small number of events occurred in the normal albumin group. This approach was chosen to reduce bias arising from sparse events and potential separation. An unadjusted model including only postoperative hypoalbuminemia and a main adjusted model including the prespecified covariates were fitted. Results are reported as odds ratios, profile-likelihood 95% confidence intervals, and *P* values.

For the secondary outcome of Clavien–Dindo grade ≥2 complications, standard binary logistic regression was used. An unadjusted model and a main adjusted model containing the same prespecified covariates were fitted, and the results are reported as odds ratios, 95% confidence intervals, and *P* values.

To assess whether the findings depended on the binary cutoff of 30 g/L, the lowest serum albumin level during postoperative days 1–7 was also analyzed as a continuous variable. Its association with Clavien–Dindo grade ≥3 and grade ≥2 complications was estimated per 5 g/L decrease, using the same covariates as in the corresponding main adjusted models. The actual sample size and number of outcome events were reported for each model. Complete-case analysis was used for variables with missing data, and no multiple imputation was performed. Descriptive analyses were based on the available data for each variable. Each regression model included only patients with complete data for all variables entered into that model, and the model-specific analytical sample size and number of outcome events were reported.

To address the potential temporal ambiguity between postoperative albumin assessment and complication occurrence, a landmark sensitivity analysis was performed using serum albumin measured on postoperative day 1 (POD1) as the exposure. Patients whose first recorded postoperative complication occurred on POD1 were excluded, and outcomes were assessed from POD2 through postoperative day 30. POD1 serum albumin was analyzed both categorically using the same 30-g/L cutoff and continuously per 5-g/L decrease. The same prespecified covariates as in the main adjusted analyses were included. Firth-penalized logistic regression was used for Clavien–Dindo grade ≥3 complications, whereas conventional binary logistic regression was used for grade ≥2 complications. Complete-case analysis was applied. This analysis was intended to reduce temporal overlap and the potential for reverse causation, although residual temporal ambiguity could not be completely excluded because exact within-day timing was unavailable.

### Subgroup analysis

In the subgroup analysis, patients with a lowest postoperative serum albumin level <30 g/L during postoperative days 1–7 were further analyzed to explore the association between albumin supplementation and Clavien-Dindo grade ≥2 complications. Propensity scores were estimated using a logistic regression model including age, sex, body mass index, American Society of Anesthesiologists physical status classification, preoperative serum albumin level, preoperative comorbidities, surgical approach, surgical method, conversion to open surgery, and intraoperative blood transfusion. The non-supplementation group was specified as the target population, and 1:1 nearest-neighbor matching without replacement was performed without a caliper, with the estimand set to the average treatment effect in the controls. Patients who were not successfully matched were excluded from the matched analysis. Covariate balance before and after matching was assessed using standardized mean differences, with an SMD <0.1 considered indicative of adequate balance. Covariate balance and propensity score overlap before and after matching were further examined using a Love plot and propensity score distribution plots.

After matching, Clavien–Dindo grade ≥2 complications within 30 days after surgery were used as the primary exploratory outcome. Conditional logistic regression stratified by matched pair was performed to assess the association between albumin supplementation and Clavien–Dindo grade ≥2 complications while accounting for the matched-pair structure. Because all covariates included in the propensity score model had absolute standardized mean differences <0.1 after matching, no additional covariates were included in the outcome model. Results are reported as conditional odds ratios with 95% confidence intervals and two-sided *P* values. This analysis was intended to evaluate the association between albumin supplementation as recorded in routine clinical practice and the outcome. It was not intended to estimate the causal treatment effect of albumin supplementation.

## Results

A total of 400 patients who underwent radical gastrectomy for gastric cancer at the First Hospital of Jilin University between January 2023 and December 2023 were initially screened. After applying the inclusion and exclusion criteria, 378 elderly patients with gastric cancer were ultimately included in the analysis. Among the 378 patients, body mass index data were missing for 11 patients, vascular invasion and perineural invasion data were each missing for 5 patients, pathological TNM stage data were missing for 4 patients, histological differentiation data were missing for 6 patients, and histological type data were missing for 3 patients. All other variables were complete. Variables with missing data were summarized and compared using the available cases. Detailed information on missing data and the model-specific analytical sample sizes is provided in Supplementary Table S1. According to the lowest serum albumin level within the first 7 postoperative days, 246 patients were classified into the hypoalbuminemia group, defined as a lowest postoperative serum albumin level <30 g/L, and 132 patients were classified into the normal albumin group, defined as a lowest postoperative serum albumin level ≥30 g/L.

The demographic, clinical, and surgery-related characteristics of the two groups are presented in Table 1. Compared with the normal albumin group, patients in the hypoalbuminemia group were older (71 [66, 75] years vs. 68 [64, 71] years, *P* < 0.001), had a lower body mass index (21.8 ± 3.3 kg/m^2^ vs. 24.0 ± 3.4 kg/m^2^, *P* < 0.001), and had a lower preoperative serum albumin level (35.6 [32.7, 37.7] g/L vs. 38.6 [37.1, 41.3] g/L, *P* < 0.001). The proportion of patients with an American Society of Anesthesiologists physical status classification of III–IV was also higher in the hypoalbuminemia group than in the normal albumin group (55% vs. 39%, *P* = 0.004).

**Table 1:** Demographic, preoperative clinical, and surgical characteristics of elderly patients undergoing radical gastrectomy stratified by early postoperative albumin level.

Variables	*N*	Overall *N* = 378	<30 *N* = 246	>=30 *N* = 132	*p*-value
BMI, Mean ± SD	367	22.6 ± 3.5	21.8 ± 3.3	24.0 ± 3.4	**<0**.**001**
Age, Median (Q1, Q3)	378	70 (65, 74)	71 (66, 75)	68 (64, 71)	**<0**.**001**
Preoperative Serum Albumin, Median (Q1, Q3)	378	36.7 (34.1, 39.2)	35.6 (32.7, 37.7)	38.6 (37.1, 41.3)	**<0**.**001**
Postoperative Length of Hospital Stay Days, Median (Q1, Q3)	378	6.0 (5.0, 8.0)	6.0 (5.0, 9.0)	5.0 (4.0, 6.0)	**<0**.**001**
Surgery Duration Hours, Median (Q1, Q3)	378	2.63 (2.15, 3.13)	2.66 (2.08, 3.12)	2.62 (2.18, 3.14)	0.965
Preoperative Neoadjuvant Chemotherapy, *n* (%)	378	6 (1.6%)	4 (1.6%)	2 (1.5%)	>0.999
Gender, *n* (%)	378				>0.999
Female		104 (28%)	68 (28%)	36 (27%)	
Male		274 (72%)	178 (72%)	96 (73%)	
ASA, *n* (%)	378				**0**.**004**
ASA I- II		192 (51%)	111 (45%)	81 (61%)	
ASA III- IV		186 (49%)	135 (55%)	51 (39%)	
Surgical Approach, *n* (%)	378				**<0**.**001**
Laparoscopic		288 (76%)	176 (72%)	112 (85%)	
Open		67 (18%)	58 (24%)	9 (6.8%)	
Robotic		23 (6.1%)	12 (4.9%)	11 (8.3%)	
Preoperative Comorbidities, *n* (%)	378	196 (52%)	119 (48%)	77 (58%)	0.082
Surgical Method, *n* (%)	378				0.066
Distal Gastrectomy		322 (85%)	203 (83%)	119 (90%)	
Proximal Gastrectomy and Total Gastrectomy		56 (15%)	43 (17%)	13 (9.8%)	
Converted to Open Surgery, *n* (%)	378	26 (6.9%)	20 (8.1%)	6 (4.5%)	0.272
Intraoperative Blood Transfusion, *n* (%)	378	48 (13%)	40 (16%)	8 (6.1%)	**0**.**007**

Bold values indicate statistically significant *p* values (*p* < 0.05).

Regarding surgery-related factors, the distribution of surgical approach differed significantly between the two groups (*P* < 0.001). The hypoalbuminemia group had a higher proportion of open surgery (24% vs. 6.8%) and a lower proportion of laparoscopic surgery (72% vs. 85%) than the normal albumin group. In addition, the proportion of patients receiving intraoperative blood transfusion was significantly higher in the hypoalbuminemia group (16% vs. 6.1%, *P* = 0.007). No statistically significant differences were observed between the two groups in sex, preoperative neoadjuvant chemotherapy, preoperative comorbidities, surgical method, conversion to open surgery, or operative time.

The pathological characteristics of the two groups are shown in Table 2. Regarding pathological characteristics, the proportions of vascular invasion (76% vs. 52%, *P* < 0.001) and perineural invasion (62% vs. 47%, *P* = 0.005) were significantly higher in the hypoalbuminemia group than in the normal albumin group. The distribution of pathological TNM stage also differed significantly between the two groups (*P* < 0.001), with a lower proportion of stage I disease (18% vs. 42%) and a higher proportion of stage III disease (54% vs. 36%) in the hypoalbuminemia group. No statistically significant differences were observed between the two groups in histological differentiation or histological type.

**Table 2:** Pathological characteristics of elderly patients undergoing radical gastrectomy stratified by early postoperative albumin level.

Variables	*N*	Overall *N* = 378	<30 *N* = 246	>=30 *N* = 132	*p*-value
Vascular Invasion, *n* (%)	373	251 (67%)	184 (76%)	67 (52%)	**<0**.**001**
Perineural Invasion, *n* (%)	373	212 (57%)	151 (62%)	61 (47%)	**0**.**005**
Pathological TNM Stage, *n* (%)	374				**<0**.**001**
Stage 1		99 (26%)	44 (18%)	55 (42%)	
Stage 2		97 (26%)	68 (28%)	29 (22%)	
Stage 3		178 (48%)	131 (54%)	47 (36%)	
Histological Differentiation, *n* (%)	372				0.140
Poorly Differentiated		121 (33%)	79 (33%)	42 (32%)	
Moderately to Poorly Differentiated		163 (44%)	113 (47%)	50 (38%)	
Moderately Differentiated and Moderately to Highly Differentiated		88 (24%)	50 (21%)	38 (29%)	
Histological Type, *n* (%)	375				0.592
Adenocarcinoma		268 (71%)	174 (71%)	94 (72%)	
Signet Ring Cell Carcinoma		31 (8.3%)	18 (7.4%)	13 (9.9%)	
Others		76 (20%)	52 (21%)	24 (18%)	

Bold values indicate statistically significant *p* values (*p* < 0.05).

### Primary outcome

Among all 378 patients, 42 developed Clavien–Dindo grade ≥3 complications within 30 days after surgery, with an overall incidence of 11.1%. Specifically, 3 of 132 patients in the normal albumin group developed Clavien–Dindo grade ≥3 complications, corresponding to an incidence of 2.3%, whereas 39 of 246 patients in the hypoalbuminemia group developed Clavien–Dindo grade ≥3 complications, corresponding to an incidence of 15.9%. The incidence of Clavien–Dindo grade ≥3 complications was significantly higher in the hypoalbuminemia group than in the normal albumin group (15.9% vs. 2.3%, *P* < 0.001).

The results of the Firth penalized logistic regression analyses are presented in Table 3. The unadjusted model included all 378 patients, among whom 42 experienced Clavien–Dindo grade ≥3 complications. Early postoperative hypoalbuminemia was associated with Clavien–Dindo grade ≥3 complications, with an unadjusted OR of 7.04 for the hypoalbuminemia group relative to the normal albumin group (95%CI, 2.63–26.20; *P* < 0.001).

**Table 3:** Association between postoperative nadir serum albumin and clavien–dindo grade ≥3 complications: firth-penalized logistic regression analysis.

Postoperative albumin status	*N*	C-D grade ≥3 complications, *n* (%)	Unadjusted OR (95% CI)	*P* value	Adjusted OR (95% CI)	*P* value	Continuous adjusted OR (95% CI)	*P* value
Albumin ≥30 g/L	132	3 (2.3%)	Ref	—	Ref	—	—	—
Albumin <30 g/L	246	39 (15.9%)	7.04 (2.63–26.20)	**<0.001**	4.51 (1.55–17.54)	**0.004**	—	—
Postoperative nadir albumin, per 5 g/L decrease	—	—	—	—	—	—	4.71 (2.43–9.51)	**<0.001**

Postoperative nadir serum albumin was defined as the lowest serum albumin level measured during postoperative days 1–7. In the categorical analysis, patients with a postoperative nadir serum albumin level ≥30 g/L served as the reference group. The unadjusted model included 378 patients, among whom 42 developed Clavien–Dindo grade ≥3 complications. The adjusted categorical model and the continuous albumin model were both based on complete-case analysis and included 363 patients, among whom 39 experienced the outcome. Both adjusted models included age, body mass index, American Society of Anesthesiologists physical status classification, preoperative serum albumin, surgical approach, surgical method, intraoperative blood transfusion, and pathological TNM stage. In the continuous albumin model, the OR represents the odds ratio for Clavien–Dindo grade ≥3 complications per 5-g/L decrease in postoperative nadir serum albumin. Firth-penalized logistic regression was used, and 95% CIs were calculated using the profile-likelihood method. All *P* values were two-sided. Full regression results for all covariates included in the main adjusted model and the continuous albumin model are provided in Supplementary Table S2.

Bold values indicate statistically significant *p* values (*p* < 0.05).

The main adjusted model was based on complete cases and included 363 patients, among whom 39 experienced Clavien–Dindo grade ≥3 complications. After adjustment for age, body mass index, American Society of Anesthesiologists physical status classification, preoperative serum albumin, surgical approach, surgical method, intraoperative blood transfusion, and pathological TNM stage, postoperative hypoalbuminemia remained associated with Clavien–Dindo grade ≥3 complications (adjusted OR, 4.51; 95%CI, 1.55–17.54; *P* = 0.004). In the same model, proximal or total gastrectomy, compared with distal gastrectomy, was also associated with Clavien–Dindo grade ≥3 complications (OR, 3.12; 95%CI, 1.42–6.69; *P* = 0.005). None of the other covariates reached statistical significance.

To assess whether this association depended on the 30-g/L cutoff, postoperative nadir serum albumin was further analyzed as a continuous variable. After adjustment for the same covariates, each 5-g/L decrease in postoperative nadir serum albumin was associated with Clavien–Dindo grade ≥3 complications (adjusted OR, 4.71; 95%CI, 2.43–9.51; *P* < 0.001). In the continuous albumin model, proximal or total gastrectomy was likewise associated with the outcome (OR, 2.80; 95%CI, 1.23–6.24; *P* = 0.015). Full results from both adjusted models are provided in Supplementary Table S2.

Overall, postoperative serum albumin levels were associated with Clavien–Dindo grade ≥3 complications within 30 days, whether albumin was modeled categorically using the 30-g/L cutoff or continuously. Because the postoperative albumin assessment window partly overlapped with the complication observation period and the temporal sequence between the albumin nadir and the first complication could not be consistently determined, these findings should be interpreted as associations rather than evidence of prediction or causality.

### Secondary outcomes

The results of the secondary outcome analysis are shown in Table 4. In the entire cohort, 184 patients developed Clavien-Dindo grade ≥2 complications within 30 days after surgery, with an overall incidence of 48.7%. Among the 132 patients in the normal albumin group, 32 developed Clavien-Dindo grade ≥2 complications, with an incidence of 24.2%; among the 246 patients in the hypoalbuminemia group, 152 developed Clavien-Dindo grade ≥2 complications, with an incidence of 61.8%. The incidence of Clavien-Dindo grade ≥2 complications was significantly higher in the hypoalbuminemia group than in the normal albumin group (61.8% vs. 24.2%, *P* < 0.001). In addition, postoperative length of hospital stay was longer in the hypoalbuminemia group than in the normal albumin group [6.0 (5.0, 9.0) d vs. 5.0 (4.0, 6.0) d, *P* < 0.001], suggesting that patients with early postoperative hypoalbuminemia may have a more complicated short-term recovery course.

**Table 4:** Association between postoperative nadir serum albumin and clavien–dindo grade ≥2 complications: logistic regression analysis.

Postoperative albumin status	*N*	C-D grade ≥2 complications, *n* (%)	Unadjusted OR (95% CI)	*P* value	Adjusted OR (95% CI)	*P* value	Continuous adjusted OR (95% CI)	*P* value
Albumin ≥30 g/L	132	32 (24.2%)	Ref	—	Ref	—	—	—
Albumin <30 g/L	246	152 (61.8%)	5.05 (3.15–8.12)	**<0.001**	3.75 (2.16–6.62)	**<0.001**	—	—
Postoperative nadir albumin, per 5 g/L decrease	—	—	—	—	—	—	4.21 (2.54–7.21)	<0.001

Postoperative nadir serum albumin was defined as the lowest serum albumin level measured during postoperative days 1–7. In the categorical analysis, patients with a postoperative nadir serum albumin level ≥30 g/L served as the reference group. The unadjusted model included 378 patients, among whom 184 developed Clavien–Dindo grade ≥2 complications. The adjusted categorical model and the continuous albumin model were both based on complete-case analysis and included 363 patients, among whom 171 experienced the outcome. Both adjusted models included age, body mass index, American Society of Anesthesiologists physical status classification, preoperative serum albumin, surgical approach, surgical method, intraoperative blood transfusion, and pathological TNM stage. In the continuous albumin model, the OR represents the odds ratio for Clavien–Dindo grade ≥2 complications per 5-g/L decrease in postoperative nadir serum albumin. Binary logistic regression was used. All *P* values were two-sided. Full regression results for all covariates included in the main adjusted model and the continuous albumin model are provided in Supplementary Table S3.

Bold values indicate statistically significant *p* values (*p* < 0.05).

The logistic regression results are presented in Table 4. The unadjusted model included all 378 patients, among whom 184 experienced Clavien–Dindo grade ≥2 complications. Postoperative hypoalbuminemia was associated with Clavien–Dindo grade ≥2 complications, with an unadjusted OR of 5.05 relative to the normal albumin group (95%CI, 3.15–8.12; *P* < 0.001).

The main adjusted model was based on complete cases and included 363 patients, among whom 171 experienced Clavien–Dindo grade ≥2 complications. After adjustment for age, body mass index, American Society of Anesthesiologists physical status classification, preoperative serum albumin, surgical approach, surgical method, intraoperative blood transfusion, and pathological TNM stage, postoperative hypoalbuminemia remained associated with Clavien–Dindo grade ≥2 complications (adjusted OR, 3.75; 95%CI, 2.16–6.62; *P* < 0.001). In this model, the adjusted OR per 1-year increase in age was 1.06 (95%CI, 1.02–1.11; *P* = 0.006). Compared with open surgery, laparoscopic surgery was associated with the outcome with an adjusted OR of 0.49 (95%CI, 0.25–0.94; *P* = 0.034). Compared with distal gastrectomy, proximal or total gastrectomy had an adjusted OR of 3.93 (95%CI, 1.99–8.16; *P* < 0.001). None of the other covariates reached statistical significance.

When postoperative nadir serum albumin was modeled continuously, each 5-g/L decrease was associated with Clavien–Dindo grade ≥2 complications after adjustment for the same covariates (adjusted OR, 4.21; 95%CI, 2.54–7.21; *P* < 0.001). In the continuous albumin model, the adjusted OR per 1-year increase in age was 1.07 (95%CI, 1.02–1.12; *P* = 0.005), and the adjusted OR for proximal or total gastrectomy compared with distal gastrectomy was 3.48 (95%CI, 1.72–7.07; *P* < 0.001). The adjusted OR for laparoscopic surgery compared with open surgery was 0.54, but this association did not reach statistical significance (95%CI, 0.27–1.06; *P* = 0.079). Full results from both adjusted models are provided in Supplementary Table S3.

Overall, postoperative albumin status was associated with Clavien–Dindo grade ≥2 complications within 30 days, whether postoperative nadir albumin was analyzed categorically or continuously. Because the albumin assessment window partly overlapped with the complication observation period and the temporal sequence could not be consistently determined, these findings should not be interpreted as predictive or causal effects.

### Sensitivity analysis

The results of the POD1 landmark sensitivity analysis are shown in Table 5. A total of 326 patients were included after excluding patients with a recorded postoperative complication on POD1 and those with missing data required for the adjusted models. Among these patients, 24 developed Clavien–Dindo grade ≥3 complications and 135 developed grade ≥2 complications from POD2 through postoperative day 30. For grade ≥3 complications, the association was attenuated when POD1 albumin was analyzed categorically (<30 vs. ≥30 g/L: adjusted OR, 1.48; 95%CI, 0.56–3.95; *P* = 0.423) or continuously (per 5-g/L decrease: adjusted OR, 1.89; 95%CI, 0.93–3.89; *P* = 0.080). For grade ≥2 complications, lower POD1 albumin remained associated with the outcome in both the categorical model (adjusted OR, 1.90; 95%CI, 1.09–3.34; *P* = 0.024) and the continuous model (adjusted OR per 5-g/L decrease, 2.08; 95%CI, 1.36–3.22; *P* < 0.001).

**Table 5:** POD1 landmark sensitivity analysis of the association between postoperative serum albumin and clavien–dindo complications.

Outcome	POD1 serum albumin status	*N*	Complications, *n* (%)	Adjusted OR (95% CI)	*P* value
C-D grade ≥3	Albumin ≥30 g/L	193	10 (5.2%)	Ref	—
	Albumin <30 g/L	133	14 (10.5%)	1.48 (0.56–3.95)	0.423
	POD1 albumin, per 5-g/L decrease	326	24 (7.4%)	1.89 (0.93–3.89)	0.080
C-D grade ≥2	Albumin ≥30 g/L	193	63 (32.6%)	Ref	—
	Albumin <30 g/L	133	72 (54.1%)	1.90 (1.09–3.34)	0.024
	POD1 albumin, per 5-g/L decrease	326	135 (41.4%)	2.08 (1.36–3.22)	<0.001

The POD1 landmark analysis included patients with valid postoperative day 1 serum albumin measurements who had no recorded postoperative complication on POD1. Outcomes were assessed from postoperative day 2 through day 30. The adjusted models included age, body mass index, American Society of Anesthesiologists physical status classification, preoperative serum albumin, surgical approach, surgical method, intraoperative blood transfusion, and pathological TNM stage. Firth-penalized logistic regression was used for Clavien–Dindo grade ≥3 complications, whereas conventional binary logistic regression was used for grade ≥2 complications. In the continuous models, the OR represents the odds ratio per 5-g/L decrease in POD1 serum albumin. The final complete-case analysis included 326 patients.

Bold values indicate statistically significant *p* values (*p* < 0.05).

### Subgroup analysis

Among patients with hypoalbuminemia, propensity score matching was further performed to evaluate the association between postoperative albumin supplementation and short-term outcomes. The hypoalbuminemia subgroup included 246 patients. Of these, 11 were excluded because of missing covariate data required for the propensity score model, leaving 235 patients for propensity score matching, including 77 patients who did not receive albumin supplementation and 158 who received albumin supplementation. After 1:1 nearest-neighbor matching without replacement, 77 matched pairs were formed, resulting in a matched cohort of 154 patients, with 77 patients in each group. All patients in the non-supplementation group were successfully matched, whereas 81 patients in the supplementation group were not included in the matched analysis. Before matching, imbalances were observed in BMI, preoperative serum albumin, intraoperative blood transfusion, and ASA classification, with absolute standardized mean differences of 0.524, 0.480, 0.125, and 0.109, respectively. The absolute standardized mean difference for the propensity score distance was 0.697. After matching, the absolute standardized mean differences for all covariates included in the propensity score model were <0.1. The largest absolute standardized mean difference among the matched covariates was 0.065, and the maximum value including the propensity score distance was 0.072.

The Love plot showed that the absolute standardized mean differences for all covariates decreased to <0.1 after matching. The propensity score distribution plots also showed improved overlap between the two groups after matching, indicating acceptable overall covariate balance. Details of the matching procedure, sample selection, and covariate balance are provided in Supplementary Table S4 and Supplementary Figure S1-S3. The demographic, preoperative clinical, and surgery-related characteristics of the matched groups are presented in Table 6, and their pathological characteristics are presented in Table 7. All variables included in the propensity score model, including age, BMI, preoperative serum albumin, sex, ASA classification, preoperative comorbidities, surgical approach, surgical method, conversion to open surgery, and intraoperative blood transfusion, met the prespecified balance criterion after matching. Operative time was longer in the albumin supplementation group than in the non-supplementation group [2.97 (2.35, 3.33) h vs. 2.60 (2.22, 3.03) h, *P* = 0.048]. In addition, postoperative length of hospital stay was longer in the albumin supplementation group [6.0 (5.0, 9.0) d vs. 5.0 (5.0, 6.0) d, *P* = 0.006]. Descriptive comparisons of vascular invasion, perineural invasion, pathological TNM stage, histological differentiation, and histological type are presented in Table 7.

**Table 6:** Demographic, preoperative, and surgery-related characteristics after propensity score matching in the hypoalbuminemia subgroup.

Variables	*N*	Overall *N* = 154	No albumin supplementation *N* = 77	Albumin supplementation *N* = 77	*p*-value
BMI, Mean ± SD	154	22.8 ± 3.1	22.8 ± 2.9	22.7 ± 3.3	0.813
Age, Median (Q1, Q3)	154	71 (66, 75)	71 (65, 75)	71 (67, 75)	0.680
Preoperative Serum Albumin, Median (Q1, Q3)	154	36.5 (34.5, 38.7)	36.6 (34.5, 38.5)	36.4 (34.6, 38.9)	0.830
Postoperative Length of Hospital Stay Days, Median (Q1, Q3)	154	6.0 (5.0, 8.0)	5.0 (5.0, 6.0)	6.0 (5.0, 9.0)	**0**.**006**
Surgery Duration Hours, Median (Q1, Q3)	154	2.79 (2.23, 3.22)	2.60 (2.22, 3.03)	2.97 (2.35, 3.33)	**0**.**048**
Preoperative Neoadjuvant Chemotherapy, *n* (%)	154	3 (1.9%)	0 (0%)	3 (3.9%)	0.245
Preoperative Comorbidities, *n* (%)	154	77 (50%)	39 (51%)	38 (49%)	>0.999
Gender, *n* (%)	154				>0.999
Female		43 (28%)	22 (29%)	21 (27%)	
Male		111 (72%)	55 (71%)	56 (73%)	
ASA, *n* (%)	154				0.628
I-II		80 (52%)	42 (55%)	38 (49%)	
III-IV		74 (48%)	35 (45%)	39 (51%)	
Surgical Approach, *n* (%)	154				0.977
Open		27 (18%)	14 (18%)	13 (17%)	
Laparoscopic		115 (75%)	57 (74%)	58 (75%)	
Robotic		12 (7.8%)	6 (7.8%)	6 (7.8%)	
Surgical Method, *n* (%)	154				>0.999
Distal		125 (81%)	63 (82%)	62 (81%)	
Prox./Total		29 (19%)	14 (18%)	15 (19%)	
Converted to Open Surgery, *n* (%)	154	12 (7.8%)	6 (7.8%)	6 (7.8%)	>0.999
Adequacy of Lymph Node Dissections, *n* (%)	154	145 (94%)	73 (95%)	72 (94%)	>0.999
Intraoperative Blood Transfusion, *n* (%)	154	11 (7.1%)	5 (6.5%)	6 (7.8%)	>0.999

Bold values indicate statistically significant *p* values (*p* < 0.05).

**Table 7:** Pathological characteristics after propensity score matching in the hypoalbuminemia subgroup.

Variables	*N*	Overall *N* = 154	No albumin supplementation *N* = 77	Albumin supplementation *N* = 77	*p*-value
Vascular Invasion, *n* (%)	153	112 (73%)	55 (71%)	57 (75%)	0.752
Perineural Invasion, *n* (%)	151	92 (61%)	47 (62%)	45 (60%)	0.948
Pathological TNM Stage, *n* (%)	152				0.935
Stage 1		33 (22%)	16 (21%)	17 (22%)	
Stage 2		44 (29%)	23 (30%)	21 (28%)	
Stage 3		75 (49%)	37 (49%)	38 (50%)	
Histological Differentiation, *n* (%)	152				0.417
Poorly Differentiated		48 (32%)	21 (28%)	27 (36%)	
Moderately to Poorly Differentiated		68 (45%)	34 (45%)	34 (45%)	
Moderately/Highly Differentiated		36 (24%)	21 (28%)	15 (20%)	
Histological Type, *n* (%)	154				0.287
Adenocarcinoma		109 (71%)	58 (75%)	51 (66%)	
Signet Ring Cell Carcinoma		13 (8.4%)	4 (5.2%)	9 (12%)	
Others		32 (21%)	15 (19%)	17 (22%)	

In the matched hypoalbuminemia subgroup, 37 of 77 patients in the non-supplementation group developed Clavien–Dindo grade ≥2 complications, corresponding to an incidence of 48.1%, whereas 54 of 77 patients in the albumin supplementation group developed Clavien–Dindo grade ≥2 complications, corresponding to an incidence of 70.1%.

The conditional logistic regression results are shown in Table 8. With the non-supplementation group as the reference, postoperative albumin supplementation was associated with Clavien–Dindo grade ≥2 complications in the propensity-score–matched cohort, with a conditional OR of 2.00 (95% CI, 1.12–3.58; *P* = 0.020). Conditional logistic regression stratified by matched pair was used to account for the matched-pair structure. Because all covariates included in the propensity score model had absolute standardized mean differences <0.1 after matching, no additional covariates were included in the outcome model.

**Table 8:** Association between postoperative albumin supplementation and clavien–dindo grade ≥2 complications in the hypoalbuminemia subgroup.

Postoperative albumin supplementation	N	CD grade ≥2 complications, *n* (%)	Conditional OR (95% CI)	*P* value
No	77	37 (48.1%)	Ref	—
Yes	77	54 (70.1%)	2.00 (1.12–3.58)	**0.020**

The no-albumin-supplementation group served as the reference group. Propensity score matching was performed using 1:1 nearest-neighbor matching without replacement. After matching, 77 patients were included in each of the albumin supplementation and no-albumin-supplementation groups. All covariates included in the propensity score model had absolute standardized mean differences <0.1 after matching; therefore, no additional covariates were included in the post-matching outcome model. Conditional logistic regression stratified by matched pair was used to account for the matched-pair structure. The model included 154 patients, of whom 91 developed Clavien–Dindo grade ≥2 complications. This result reflects the association between postoperative albumin supplementation and complications and should not be used to draw causal conclusions regarding the benefit or harm of albumin supplementation.

Bold values indicate statistically significant *p* values (*p* < 0.05).

Overall, in the propensity-score–matched hypoalbuminemia subgroup, patients who received postoperative albumin supplementation did not have a lower incidence of Clavien–Dindo grade ≥2 complications, and albumin supplementation was not associated with fewer complications. Albumin supplementation was not randomly assigned, and the supplementation window during postoperative days 1–7 partly overlapped with the 30-day complication observation period. Therefore, the temporal sequence between albumin supplementation and the first occurrence of a complication could not be consistently determined. Some patients may have received albumin supplementation because of poor postoperative recovery or an early complication. Accordingly, the observed association may have been affected by confounding by indication, residual confounding, and reverse causation. These findings reflect an association between albumin supplementation and complications and should not be interpreted as evidence of the efficacy or harm of albumin supplementation.

## Discussion

This study systematically evaluated the associations of postoperative hypoalbuminemia and albumin supplementation with short-term complications in elderly patients with gastric cancer. The results showed that postoperative serum albumin was associated with both Clavien–Dindo grade ≥3 and grade ≥2 complications within 30 days after surgery, whether the lowest serum albumin level during postoperative days 1–7 was categorized using the 30 g/L cutoff or analyzed as a continuous variable. In the hypoalbuminemia subgroup, patients who received albumin supplementation did not have a lower incidence of Clavien–Dindo grade ≥2 complications. Because albumin assessment, albumin supplementation, and some complications occurred within overlapping postoperative time windows, these findings should be interpreted as concurrent associations and cannot establish temporal sequence, predictive performance, or causal effects.

Several previous retrospective studies have reported that hypoalbuminemia is associated with an increased risk of postoperative complications, including infection, pneumonia, anastomotic leakage, and venous thromboembolic events (7, 9, 21). In the present study, postoperative hypoalbuminemia was associated with both Clavien–Dindo grade ≥3 and grade ≥2 complications, and statistical associations were observed when postoperative albumin was analyzed as either a categorical or continuous variable. These findings are broadly consistent with previous studies, although the relationship between hypoalbuminemia and complications is unlikely to represent a simple unidirectional causal process. The decline in serum albumin after radical gastrectomy is multifactorial and often cannot be fully explained by blood loss or fluid dilution. Surgical stress and inflammation, endothelial glycocalyx injury, increased capillary permeability, and transvascular albumin escape may all contribute to lower serum albumin levels. At the same time, these processes may aggravate tissue edema and impair postoperative recovery, thereby providing a shared pathophysiological basis for hypoalbuminemia and postoperative complications (22–25). In addition, the reduction in colloid osmotic pressure, interstitial fluid retention, and impairment of the transport and antioxidant functions associated with hypoalbuminemia may further compromise wound healing and recovery of organ function (26). Conversely, early postoperative infection, anastomosis-related complications, or persistent exudation that has not yet been recognized may intensify inflammation and protein loss and thereby further reduce serum albumin levels. Therefore, the observed associations may reflect a combination of shared pathophysiological processes, the potential adverse effects of hypoalbuminemia, and reverse causation.

Importantly, the POD1 landmark sensitivity analysis provided additional information regarding this temporal issue. When serum albumin measured on POD1 was used as the exposure and patients with recorded complications on POD1 were excluded, the estimated associations were substantially attenuated. The associations with Clavien–Dindo grade ≥2 complications remained statistically significant, whereas those with grade ≥3 complications were no longer statistically significant. These findings suggest that part of the stronger association observed using the POD1–7 nadir albumin may reflect overlapping postoperative processes and potential reverse causation. Nevertheless, the direction of association remained consistent across all sensitivity models, and residual temporal ambiguity cannot be fully excluded because exact within-day timing was unavailable.

This process may be particularly pronounced in elderly patients undergoing radical gastrectomy for gastric cancer. D2 lymphadenectomy for gastric cancer involves extensive dissection around perigastric vessels and along the superior border of the pancreas. Postoperative peritoneal wound exudation, altered lymphatic drainage, and intestinal wall edema after gastrointestinal reconstruction may all promote the movement of albumin-rich fluid into the interstitial space or abdominal cavity, thereby aggravating third-space fluid accumulation (27). At the same time, elderly patients often have reduced nutritional reserves, a greater burden of comorbidities, and impaired physiological compensatory capacity, making them more vulnerable to perioperative inflammatory responses and volume shifts. Therefore, postoperative hypoalbuminemia is more likely to represent an integrated perioperative manifestation of surgical trauma, inflammation, altered vascular permeability, and fluid management. It may serve as an important risk indicator for identifying patients at higher risk of postoperative complications rather than merely representing a nutritional abnormality.

In addition to early postoperative hypoalbuminemia, several clinical and surgical factors were associated with postoperative complications, although the findings were not entirely consistent across outcome models. Proximal or total gastrectomy was associated with both Clavien–Dindo grade ≥2 and grade ≥3 complications, suggesting that surgical method and the complexity of gastrointestinal reconstruction may be important factors affecting postoperative outcomes. Age was associated with Clavien–Dindo grade ≥2 complications in both the categorical and continuous albumin models. Compared with open surgery, laparoscopic surgery reached statistical significance only in the categorical albumin model and not in the continuous albumin model; therefore, the finding for surgical approach should be interpreted cautiously. These differences may partly reflect the relatively limited number of Clavien–Dindo grade ≥3 events, which reduced the statistical power to identify associations involving some covariates. They also suggest that, compared with grade ≥3 complications, the broader category of grade ≥2 complications may be more readily influenced by patients’ baseline condition, surgical method, and local tumor characteristics.

Compared with distal gastrectomy, proximal or total gastrectomy generally involves more extensive resection, more complex gastrointestinal reconstruction, and greater technical demands for anastomosis. Therefore, the observed associations of proximal or total gastrectomy with both complication outcomes are clinically plausible. The findings for age and surgical approach suggest that physiological reserve and surgery-related factors may jointly influence postoperative recovery (28, 29).

Postoperative complications may also have implications beyond short-term recovery. Kamarajah et al. reported that severe anastomotic leakage after gastrectomy was associated with worse long-term overall survival, whereas its association with recurrence-free survival was not statistically significant (30). More recently, Kim et al. suggested that persistent systemic inflammation may partly mediate the association between postoperative complications and long-term survival after gastric cancer surgery (31). However, the present study was designed to evaluate 30-day postoperative outcomes and did not systematically collect long-term survival or recurrence data; therefore, these relationships could not be examined in our cohort.

In the subgroup of patients with hypoalbuminemia, this study further explored the association between postoperative albumin supplementation and short-term outcomes. Patients who received postoperative albumin supplementation did not have a lower incidence of complications; however, this observation cannot be used to directly evaluate the therapeutic effect of albumin supplementation. Operative time was not included in the propensity score model and remained different between the matched groups; therefore, residual confounding related to surgical complexity cannot be completely excluded. Previous studies, including the SAFE trial, the SOAP study, and the more recent SuperAdd trial, have likewise not demonstrated consistent clinical benefits of albumin administration in critically ill or perioperative populations (10, 11, 32). Several explanations may account for this finding. From a pathophysiological perspective, if vascular barrier dysfunction has not been restored, exogenously infused albumin may leak into the interstitial space together with endogenous albumin, which could theoretically aggravate tissue edema and increase the risk of pulmonary complications (4). In a highly inflammatory and oxidative stress environment, albumin molecules may undergo oxidative modifications, particularly at the Cys-34 site, resulting in marked impairment of their antioxidant, drug-binding, and transport functions (26). Therefore, even if albumin infusion increases the serum albumin concentration to ≥30 g/L, the effective biological function of albumin may not recover synchronously (33). In addition, confounding by indication is an important issue in the present study. Although propensity score matching improved the balance of measured covariates, unmeasured confounding could not be completely excluded. In clinical practice, physicians may be more likely to administer albumin to patients with more severe illness, greater surgical trauma, or unsatisfactory postoperative recovery. Thus, in retrospective data, albumin administration may accompany an unfavorable prognosis rather than cause it. Some patients may also have received albumin supplementation only after a complication or poor postoperative recovery had already occurred. Therefore, the observed association cannot be used to determine the true efficacy or harm of albumin supplementation and is more likely to reflect the complex relationships among perioperative clinical status, treatment selection, and short-term outcomes.

The incremental value of the present study lies not in once again demonstrating a general association between albumin-related measures and postoperative complications after gastric cancer surgery, but in refining the study perspective and exposure definition. Previous gastric cancer studies have mainly focused on preoperative albumin and albumin-based nutritional indices or have described perioperative albumin changes using absolute or relative decreases from preoperative to postoperative levels, albumin ratios, or measurements obtained at selected postoperative time points. In contrast, the present study focused on the postoperative hypoalbuminemic state itself in elderly patients undergoing radical gastrectomy. Patients were classified according to the lowest serum albumin level during postoperative days 1–7 using the clinically applied absolute cutoff of 30 g/L, and associations with both Clavien–Dindo grade ≥3 and grade ≥2 complications were evaluated. Compared with preoperative measures or perioperative change-based measures, this definition more directly corresponds to the postoperative hypoalbuminemic state encountered by clinicians and helps characterize its relationship with the overall burden of complications in this specific population. In addition, the study explored the association between albumin supplementation as delivered in routine clinical practice and short-term outcomes among patients with hypoalbuminemia. This analysis was intended to describe actual clinical supplementation behavior rather than evaluate the causal treatment effect of albumin supplementation.

Previous studies of albumin and clinical outcomes have included highly heterogeneous populations, such as patients with cirrhosis, sepsis, multiple trauma, intensive care unit admission, or different types of major surgery (10, 32, 34–36). The predominant mechanisms of hypoalbuminemia differ among these underlying conditions. In cirrhosis, reduced hepatic synthesis is usually predominant; severe infection is frequently accompanied by increased catabolism and inflammation-mediated capillary leakage; and acute postoperative hypoalbuminemia is more strongly influenced by increased vascular permeability, third-space fluid shifts, and perioperative hemodilution. Failure to distinguish adequately among these pathophysiological backgrounds may dilute population-specific associations and may even produce inconsistent findings across studies.

The present findings suggest that early postoperative hypoalbuminemia may serve as an important indicator for identifying patients at higher risk of complications. For patients with a postoperative serum albumin level <30 g/L, clinical management should not focus solely on correcting the laboratory value. Surgical trauma, inflammatory response, perioperative fluid balance, and the overall postoperative course should also be assessed, with particular attention to infection, anastomosis-related complications, or other causes of poor recovery. Closer postoperative monitoring and targeted examinations may be considered when clinically indicated. At the same time, this study did not observe an association between albumin supplementation and a lower incidence of complications. Because some patients may have received albumin only after an early complication or poor recovery had developed, this finding may be affected by confounding by indication and reverse causation. Therefore, the study neither demonstrates that albumin supplementation improves outcomes nor establishes that it is ineffective or increases complication risk. The findings are insufficient to support the use of postoperative serum albumin <30 g/L as an isolated indication for albumin supplementation. Decisions regarding albumin administration should remain individualized according to circulatory status, volume status, ongoing protein loss, organ function, and the overall postoperative course. The clinical implication of this study is not to propose a new fixed transfusion threshold, but to regard postoperative hypoalbuminemia as a signal for comprehensive assessment and risk stratification and to shift postoperative management from simple numerical correction toward systematic evaluation of underlying causes and overall recovery.

Nevertheless, several limitations of this study should be acknowledged. As a single-center retrospective study, the sample size and number of outcome events were limited. Although propensity score matching and multivariable adjustment were used to reduce between-group differences, confounding by indication could not be completely eliminated. Patients who received albumin therapy may have had unrecorded clinical features indicating greater baseline severity, which might have masked the potential benefits of albumin supplementation. In addition, the dose, timing, and indications for albumin supplementation were mainly determined by the attending physicians, and no unified treatment protocol was applied. This limited our ability to determine whether there may be a specific beneficial window related to albumin dose or timing. Furthermore, the lowest serum albumin level and albumin supplementation during postoperative days 1–7 were used as exposures, whereas complications were observed for 30 days after surgery. Therefore, the exposure assessment window partly overlapped with the outcome observation period. The retrospective records did not consistently provide the precise temporal sequence among the albumin nadir, the first albumin supplementation, and the first occurrence of a complication. Some patients may have reached their albumin nadir or received albumin supplementation only after a complication or poor recovery had already developed. Consequently, the temporal relationship between exposure and outcome could not be fully established, and reverse causation could not be excluded. These results should therefore be interpreted as associations and should not be used for strict prognostic inference or causal evaluation of albumin supplementation. Long-term survival and tumor recurrence were not systematically collected in the present dataset; therefore, the potential associations of postoperative hypoalbuminemia and postoperative complications with long-term oncological outcomes could not be assessed. Future prospective randomized controlled trials with clearly defined supplementation indications are needed to identify the patient populations and timing for which albumin supplementation may be appropriate and to determine its effects on clinical outcomes.

## Conclusion

A postoperative nadir serum albumin level <30 g/L during postoperative days 1–7 was associated with both Clavien–Dindo grade ≥3 and grade ≥2 complications within 30 days after radical gastrectomy in elderly patients with gastric cancer. In the POD1 landmark sensitivity analysis, the estimated associations were attenuated; associations with grade ≥2 complications remained statistically significant, whereas those with grade ≥3 complications were no longer statistically significant. These findings indicate that the temporal relationship between postoperative hypoalbuminemia and complications is complex and further support interpreting the main results as associations rather than predictive or causal effects. Postoperative hypoalbuminemia may reflect perioperative changes related to surgical trauma, inflammation, and fluid redistribution, but may also be influenced by early complications and poor postoperative recovery. Therefore, it may be more appropriately regarded as a clinical signal accompanying a complex postoperative course and a greater burden of complications rather than as a strict predictive marker. In patients who develop postoperative hypoalbuminemia, further assessment should include inflammatory status, fluid balance, ongoing protein loss, overall recovery, and possible underlying complications.

In the propensity-score–matched hypoalbuminemia subgroup, recorded postoperative albumin supplementation was not associated with a lower incidence of complications. However, because albumin supplementation was not randomly assigned and some patients may have received it only after an early complication or poor recovery had already developed, this observation may have been affected by confounding by indication and reverse causation. It cannot be used to determine the efficacy or harm of albumin supplementation. The specific threshold and clinical indications for postoperative albumin administration require further evaluation in prospective studies.

## Data Availability

The data analyzed in this study contain patients’ clinical information and are subject to restrictions related to patient privacy protection and institutional data management policies; therefore, they are not publicly available. De-identified data may be made available upon reasonable request, subject to review and approval by the corresponding author and the institution and provided that all applicable ethical approval and data management requirements are met. The R code used for the main statistical analyses is also available from the corresponding author upon reasonable request.
