# Supplementary material for: Postoperative hypoalbuminemia, albumin supplementation, and short-term complications after radical gastrectomy in elderly gastric cancer patients
Source: Front Med (Lausanne). 2026 Oct 28;13:1978269. doi: 10.3389/fmed.2026.1978269 (PMC13752807; doi:10.3389/fmed.2026.1978269)
Supplement: Supplementary file 1 [file Table1.docx]

**Supplementary Table S1. Missing data and model-specific analytical sample sizes**

**Panel A. Variable-specific missingness**

| **Variable** | **Total sample, n** | **Available data, n** | **Missing data, n** | **Missing percent, %** |
| --- | --- | --- | --- | --- |
| BMI | 378 | 367 | 11 | 2.9% |
| Age | 378 | 378 | 0 | 0.0% |
| Preoperative Serum Albumin | 378 | 378 | 0 | 0.0% |
| Postoperative Length of Hospital Stay Days | 378 | 378 | 0 | 0.0% |
| Surgery Duration Hours | 378 | 378 | 0 | 0.0% |
| Preoperative Neoadjuvant Chemotherapy | 378 | 378 | 0 | 0.0% |
| Gender | 378 | 378 | 0 | 0.0% |
| ASA | 378 | 378 | 0 | 0.0% |
| Surgical Approach | 378 | 378 | 0 | 0.0% |
| Preoperative Comorbidities | 378 | 378 | 0 | 0.0% |
| Surgical Method | 378 | 378 | 0 | 0.0% |
| Converted to Open Surgery | 378 | 378 | 0 | 0.0% |
| Intraoperative Blood Transfusion | 378 | 378 | 0 | 0.0% |
| Vascular Invasion | 378 | 373 | 5 | 1.3% |
| Perineural Invasion | 378 | 373 | 5 | 1.3% |
| Pathological TNM Stage | 378 | 374 | 4 | 1.1% |
| Histological Differentiation | 378 | 372 | 6 | 1.6% |
| Histological Type | 378 | 375 | 3 | 0.8% |
| Postoperative serum albumin nadir | 378 | 378 | 0 | 0.0% |
| C-D grade ≥3 complications | 378 | 378 | 0 | 0.0% |
| C-D grade ≥2 complications | 378 | 378 | 0 | 0.0% |

Note: Complete-case analysis was used for variables with missing data, and no multiple imputation was performed. Model-specific analytical samples included patients with complete data for all variables entered into the corresponding model. The 15 patients excluded from the adjusted models comprised 11 patients with missing BMI data and 4 patients with missing pathological TNM-stage data.

**Panel B. Model-specific analytical sample sizes, outcome events, and exclusions due to missing data**

| **Outcome** | **Method** | **Model** | **Analysis sample, n** | **Outcome events, n** | **Excluded because of missing covariate data, n** |
| --- | --- | --- | --- | --- | --- |
| C-D grade ≥3 complications | Firth penalized logistic regression | Unadjusted categorical-albumin model | 378 | 42 | 0 |
| C-D grade ≥3 complications | Firth penalized logistic regression | Main adjusted categorical-albumin model | 363 | 39 | 15 |
| C-D grade ≥3 complications | Firth penalized logistic regression | Adjusted continuous-albumin model, per 5-g/L decrease | 363 | 39 | 15 |
| C-D grade ≥2 complications | Logistic regression | Unadjusted categorical-albumin model | 378 | 184 | 0 |
| C-D grade ≥2 complications | Logistic regression | Main adjusted categorical-albumin model | 363 | 171 | 15 |
| C-D grade ≥2 complications | Logistic regression | Adjusted continuous-albumin model, per 5-g/L decrease | 363 | 171 | 15 |
