# Supplementary material for: Postoperative hypoalbuminemia, albumin supplementation, and short-term complications after radical gastrectomy in elderly gastric cancer patients
Source: Front Med (Lausanne). 2026 Oct 28;13:1978269. doi: 10.3389/fmed.2026.1978269 (PMC13752807; doi:10.3389/fmed.2026.1978269)
Supplement: Supplementary file 2 [file Table2.docx]

**Supplementary Table S2. Full results of multivariable Firth penalized logistic regression analyses for Clavien–Dindo grade ≥3 complications**

**Panel A. Model with postoperative albumin status as a categorical variable**

| **Variable** | **Level** | **OR (95% CI)** | **P value** |
| --- | --- | --- | --- |
| Postoperative albumin minimum Value | Postoperative Alb≥30 | Ref |  |
|  | Postoperative Alb ＜30 | 4.51 (1.55-17.54) | **0.004** |
| Age |  | 1.02 (0.96-1.09) | 0.455 |
| BMI |  | 0.96 (0.86-1.07) | 0.428 |
| Preoperative albumin, per 1 g/L |  | 0.99 (0.90-1.09) | 0.860 |
| ASA | ASA I-II | Ref |  |
|  | ASA III- IV | 1.14 (0.53-2.48) | 0.733 |
| Surgical Approach | Open | Ref |  |
|  | Laparoscopic | 0.63 (0.29-1.41) | 0.249 |
|  | Robotic | 0.68 (0.11-3.09) | 0.637 |
| Surgical Method | Distal gastrectomy | Ref |  |
|  | Proximal Gastrectomy and Total Gastrectomy | 3.12 (1.42-6.69) | **0.005** |
| Intraoperative Blood Transfusion | No | Ref |  |
|  | Yes | 1.81 (0.69-4.43) | 0.220 |
| Pathological TNM stage | Stage 1 | Ref |  |
|  | Stage 2 | 1.06(0.39-3.05) | 0.910 |
|  | Stage 3 | 0.92 (0.37-2.48) | 0.861 |

**Panel B. Postoperative albumin nadir modeled as a continuous variable (per 5-g/L decrease)**

| **Variable** | **Level** | **OR (95% CI)** | **P value** |
| --- | --- | --- | --- |
| Postoperative albumin nadir, per 5 g/L decrease |  | 4.71 (2.43-9.51) | **<0.001** |
| Age |  | 1.02 (0.96-1.09) | 0.497 |
| BMI |  | 0.98 (0.88-1.10) | 0.787 |
| Preoperative albumin, per 1 g/L |  | 1.05 (0.96-1.17) | 0.294 |
| ASA | ASA I-II | Ref |  |
|  | ASA III- IV | 0.99 (0.45-2.18) | 0.990 |
| Surgical Approach | Open | Ref |  |
|  | Laparoscopic | 0.63 (0.28-1.47) | 0.279 |
|  | Robotic | 0.62 (0.10-2.93) | 0.562 |
| Surgical Method | Distal gastrectomy | Ref |  |
|  | Proximal Gastrectomy and Total Gastrectomy | 2.80 (1.23-6.24) | **0.015** |
| Intraoperative Blood Transfusion | No | Ref |  |
|  | Yes | 2.06 (0.78-5.18) | 0.141 |
| Pathological TNM stage | Stage 1 | Ref |  |
|  | Stage 2 | 1.10 (0.39-3.25) | 0.864 |
|  | Stage 3 | 0.77 (0.29-2.16) | 0.604 |

**Note:** Odds ratios and 95% confidence intervals were estimated using Firth penalized logistic regression. Both models were adjusted for age, BMI, preoperative serum albumin, ASA class, surgical approach, extent of gastrectomy, intraoperative blood transfusion, and pathological TNM stage. The continuous albumin estimate represents the association per 5-g/L decrease in the postoperative albumin nadir. Ref, reference category; ASA, American Society of Anesthesiologists; BMI, body mass index; CI, confidence interval; OR, odds ratio.
