# Supplementary material for: Postoperative hypoalbuminemia, albumin supplementation, and short-term complications after radical gastrectomy in elderly gastric cancer patients
Source: Front Med (Lausanne). 2026 Oct 28;13:1978269. doi: 10.3389/fmed.2026.1978269 (PMC13752807; doi:10.3389/fmed.2026.1978269)
Supplement: Supplementary file 4 [file Table4.docx]

**Supplementary Table S4. Propensity-score matching specifications, sample selection, and covariate balance**

**Panel A. Propensity-score matching specifications and matching results**

| **Item** | **Value** |
| --- | --- |
| Covariates included in the propensity-score model | Age, sex, BMI, ASA class, preoperative serum albumin, preoperative comorbidities, surgical approach, extent of gastrectomy, conversion to open surgery, and intraoperative blood transfusion |
| Matching method | Nearest-neighbor matching |
| Propensity-score estimation method | Logistic regression |
| Matching ratio | 1:1 |
| Replacement | No |
| Matching estimand | ATC |
| Caliper | None |
| No albumin supplementation before matching, n | 77 |
| Albumin supplementation before matching, n | 158 |
| No albumin supplementation after matching, n | 77 |
| Albumin supplementation after matching, n | 77 |
| Unmatched patients in the no-supplementation group, n | 0 |
| Unmatched patients in the supplementation group, n | 81 |
| Matched pairs, n | 77 |
| Maximum post-matching absolute SMD among matching covariates | 0.065 |
| Maximum post-matching absolute SMD including propensity score distance | 0.072 |
| All post-matching absolute SMDs <0.1 | Yes |

Note: ATC, average treatment effect in controls. Analyses were performed using R version 4.4.2, with MatchIt version 4.7.2 for propensity-score matching and cobalt version 4.6.3 for balance assessment.

**Panel B. Complete-case sample selection for propensity-score matching**

| **Item** | **N** |
| --- | --- |
| Low-albumin subgroup before PSM covariate complete-case filtering | 246 |
| Complete PSM covariate sample | 235 |
| Excluded before PSM because of missing PSM covariates | 11 |

Note: Variable-specific missingness is reported in Supplementary Table S1. Patients with missing data for any propensity-score covariate were excluded using complete-case analysis; no multiple imputation was performed.

**Panel C. Covariate balance before and after propensity-score matching**

| **Covariate** | **Absolute SMD before matching** | **Absolute SMD after matching** |
| --- | --- | --- |
| Propensity score distance | 0.697 | 0.072 |
| BMI, kg/m² | 0.524 | 0.041 |
| Preoperative Serum Albumin, g/L | 0.480 | 0.001 |
| Intraoperative Blood Transfusion | 0.125 | 0.013 |
| ASA group III-IV | 0.109 | 0.052 |
| Age | 0.061 | 0.065 |
| Surgical Approach: open | 0.059 | 0.013 |
| Surgical Approach: laparoscopic | 0.019 | 0.013 |
| Surgical Approach: robotic | 0.040 | 0.000 |
| Preoperative Comorbidities | 0.044 | 0.013 |
| Converted to Open Surgery | 0.011 | 0.000 |
| Gender: Male | 0.007 | 0.013 |
| Surgical Method group: proximal/total | 0.002 | 0.013 |

Note: Values are presented as absolute standardized mean differences. An absolute standardized mean difference of <0.1 was considered indicative of adequate covariate balance. For binary covariates, only one category is presented because the complementary category has the same absolute standardized mean difference. For categorical covariates with more than two levels, balance is presented separately for each category.
